# Supplementary material for: Unveiling genetic signatures of immune response in immune-related diseases through single-cell eQTL analysis across diverse conditions
Source: Nat Commun. 2025 Aug 4;16:7134. doi: 10.1038/s41467-025-61192-4 (PMC12322060; doi:10.1038/s41467-025-61192-4)
Supplement: Supplementary file 1 — Supplementary Information [file 41467_2025_61192_MOESM1_ESM.pdf]

Supplementary Figure 1

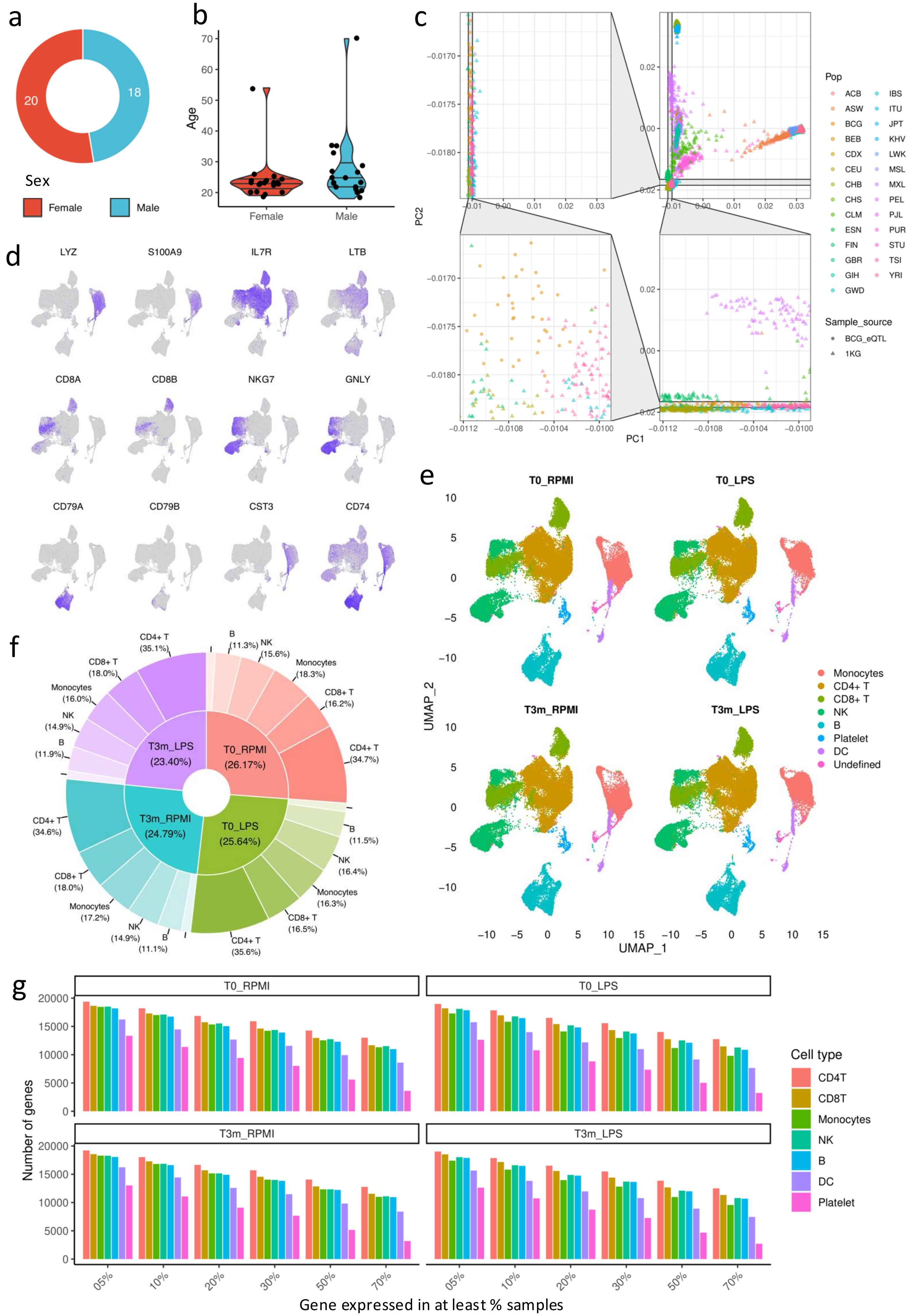

Supplementary Figure 1

(a & b) Sex and age distributions of the cohort. (n = 32 individuals)

(c) PCA plot displays the genetic relationship between the current study cohort and 1000 genome cohort. The colors of the markers suggest sub-populations and the shape of the markers indicate the source of the donors.

(d) Cell types identified from each condition.

(e) Expression of cell lineage markers genes. N = 186,341 cells

(f) Proportion of major cell lineages from each condition.

(g) Number (y-axis) of expressed genes per cell type (per colored bar) per condition (per panel) at different threshold (x-axis).

Supplementary Figure 2

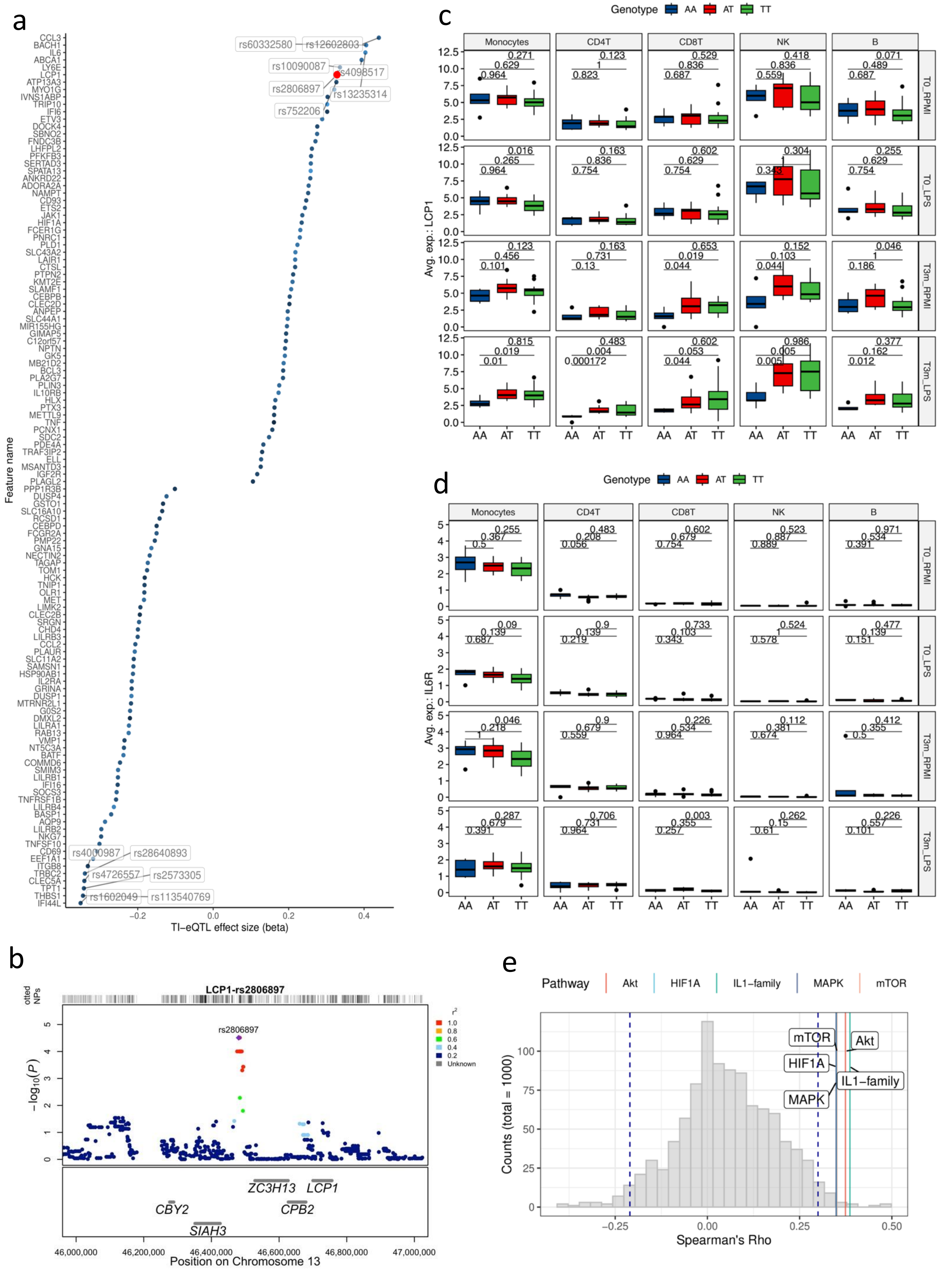

Supplementary Figure 2

(a) The overview of eQTL effects of previously identified TI genes. The x-axis is the effect size and the y axis is the corresponding eGene. Top eVariant (|beta| >= 0.3) were labeled with the rsID and the example TI eQTL *LCP1*-rs2806897 were colored in red. The eQTL are estimated using linear mixed model adjusting age and gender, n = 152 samples.

(b) *LCP1* locus that was prioritized by TI eQTL analysis.

(c) Boxplot showing expression levels of *LCP1* per genotype per cell type per condition. In box-pots, whiskers expand to minima and maxima, boxes indicate 25th and 75th percentiles, centers represent medians. N = 152 samples.

(d) Boxplot showing expression of *IL6R* per genotype per cell type per condition. In box-pots, whiskers expand to minima and maxima, boxes indicate 25th and 75th percentiles, centers represent medians. N = 152 samples.

(e) Histogram plot showing the permutation test results for pathway scores.

Supplementary Figure 3

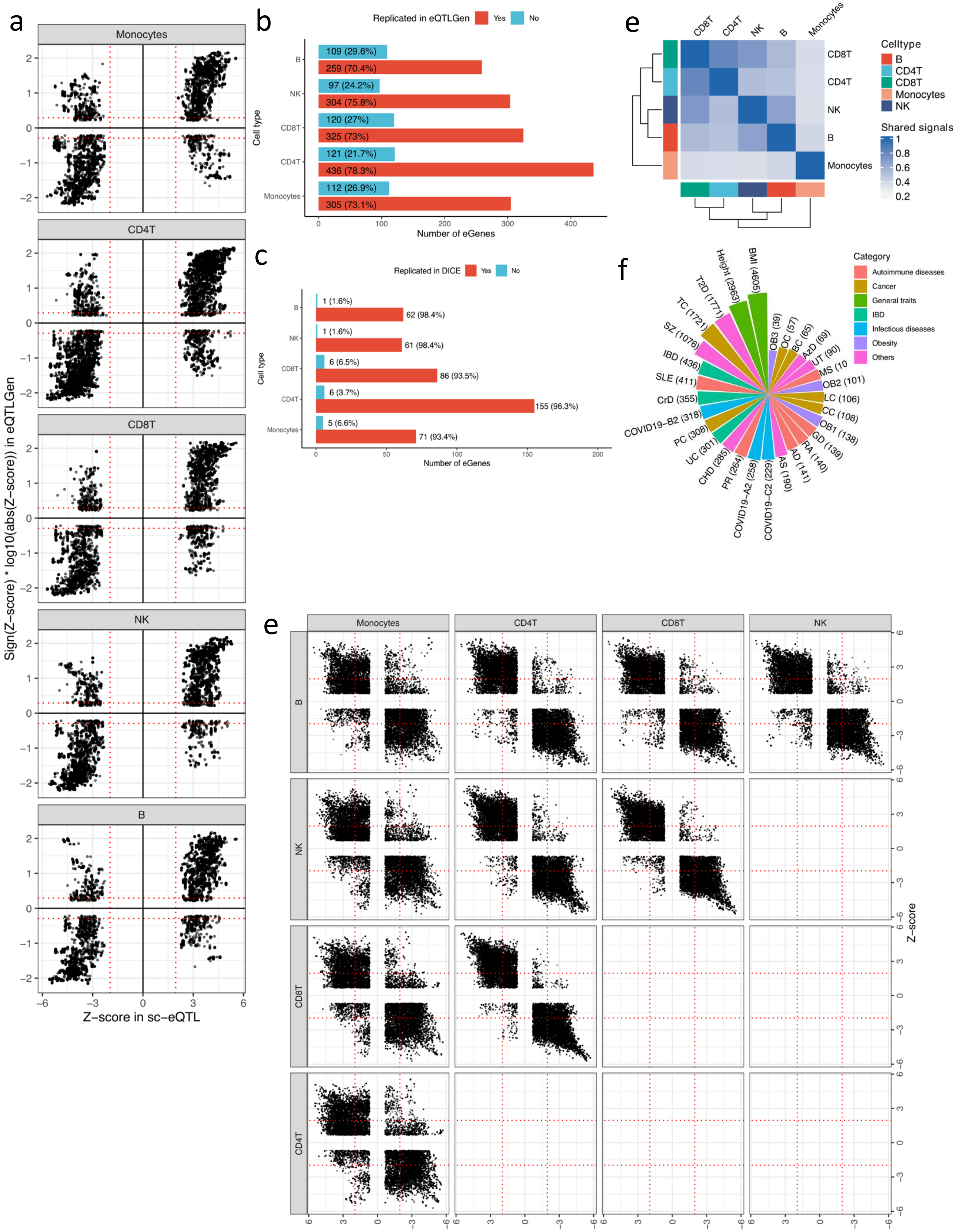

Supplementary Figure 3

(a) Concordance between consistent sc-eQTL and eQTL from eQTLGen.

(b) Number of eGenes (consistent eQTL analysis) that are replicated in eQTLGen.

(c) Number of eGenes (consistent eQTL analysis) that are replicated in DICE database[3].

(d) Shared eQTL signals among cell lineages in consistent eQTL analyses.

(e) Concordance of consistent eQTL across major cell lineages.

(f) Number of independent loci identified from each publicly available GWAS summary statistics.

Supplementary Figure 4

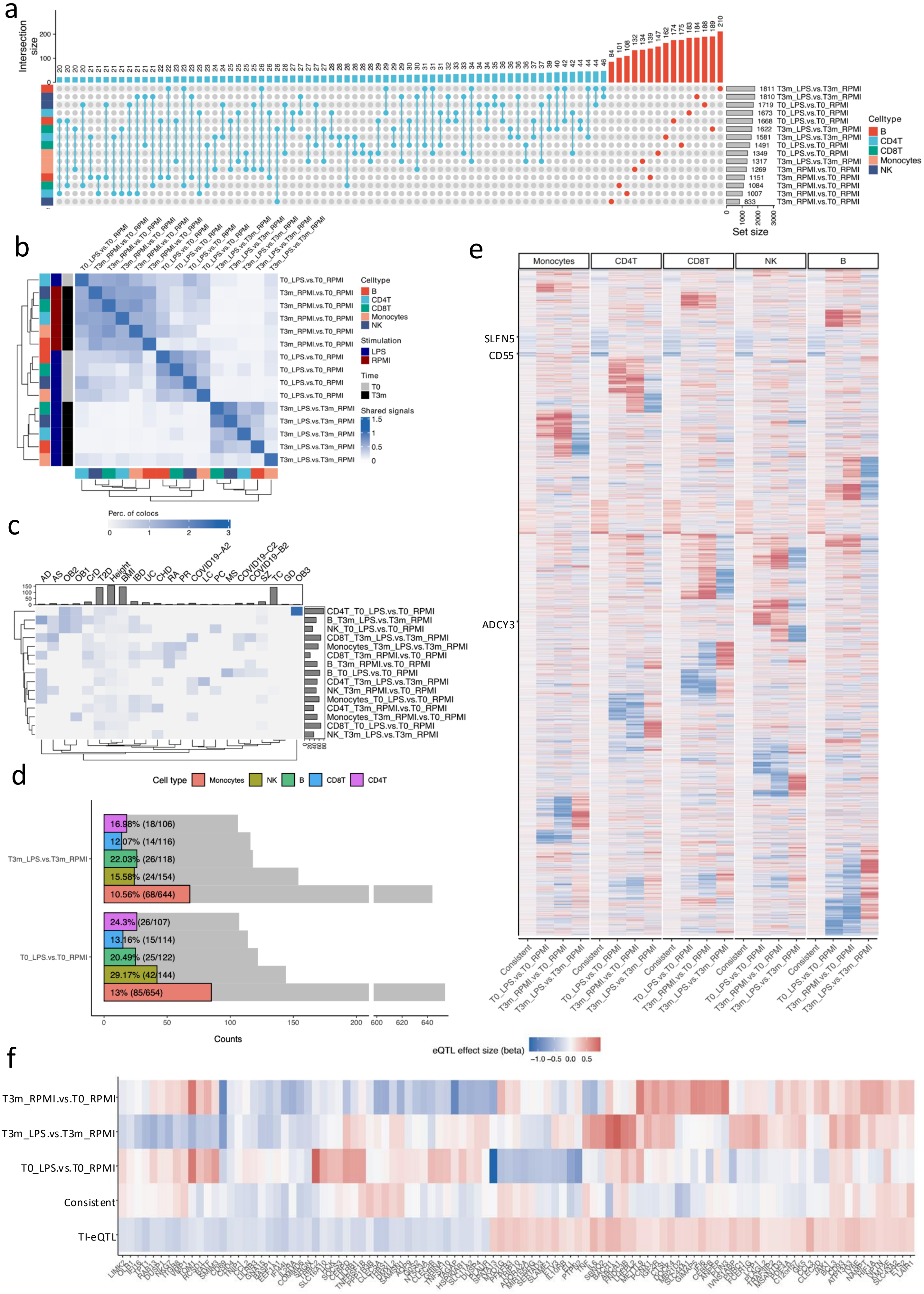

Supplementary Figure 4

(a) Number eGenes identified in response eQTL analyses.

(b) Shared eQTL signals across cell lineages and conditions, which were estimated in response eQTL analysis.

(c) Colocalization analysis between response eQTL and 23 publicly available GWAS summary statistics.

(d) DE genes that were also identified as eGenes upon perturbations. The x-axis is the number of genes (eGene or DE genes) while the y-axis is the conditions. The color of the bar indicates cell types and the labels are percentage (exact number) of DE genes that were also identified as eGenes.

(e) Heatmap displaying the eQTL effects of identified sc-eQTLs. The x-axis represents the conditions for each cell type, while the y-axis lists eQTLs that were significant in at least one condition from one cell type. The colors indicate eQTL effects (beta coefficients). Additionally, example eQTLs are labelled along the y-axis.

(f) Comparison of eQTL effects for the identified TI response genes. The x-axis represents the names of TI gene, while y-axis indicates the eQTL conditions. The colors indicate the effect sizes of the eQTLs.

Supplementary Figure 5

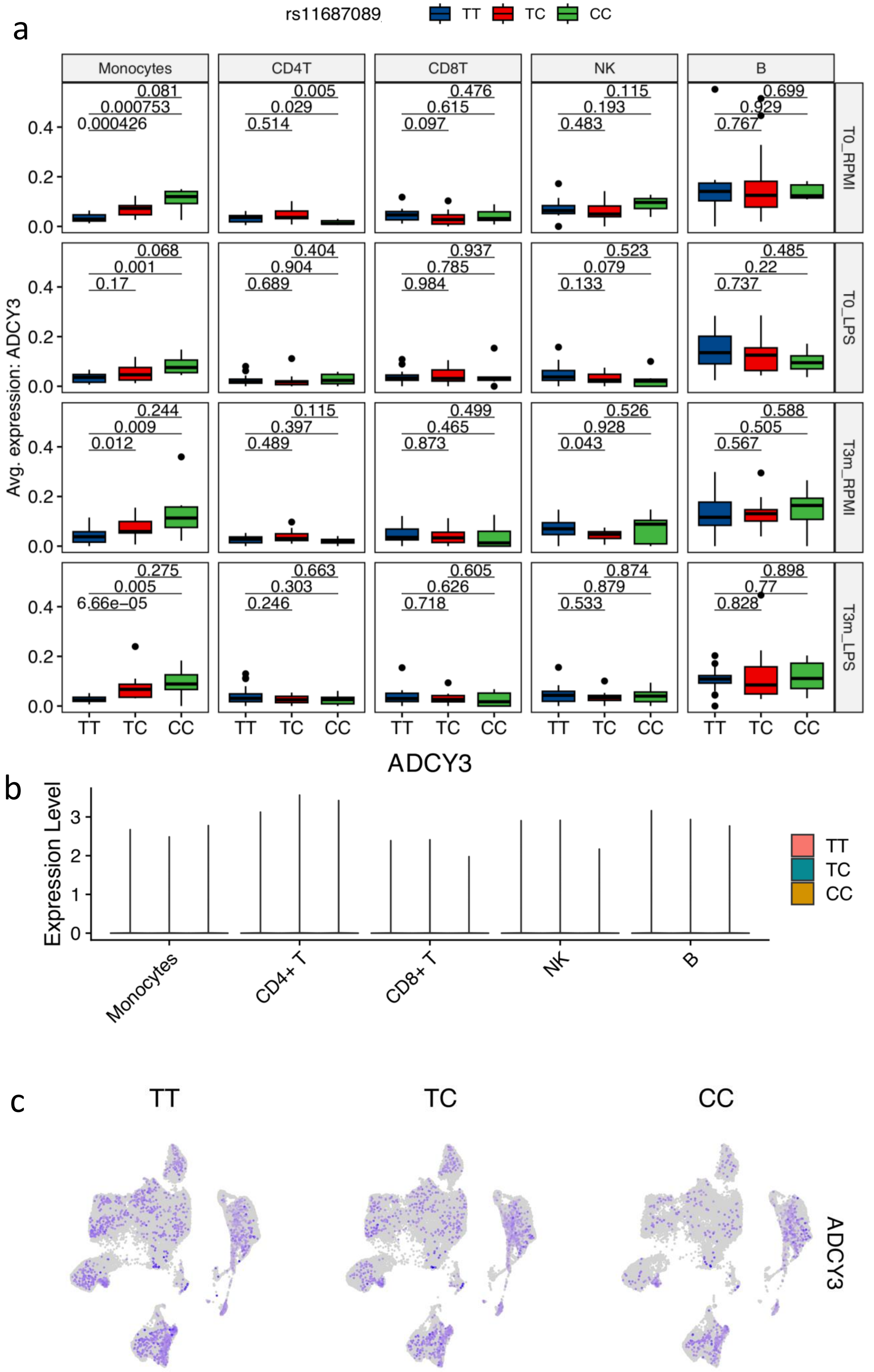

Supplementary Figure 5

(a) eQTL effects of *ADCY3* locus estimated per condition per cell lineage type. In box-pots, whiskers expand to minima and maxima, boxes indicate 25th and 75th percentiles, centers represent medians.

(b) Violin plot displays the eQTL effects of *ADCY3* locus at single-cell level. N = 186,341 cells

(c) Expression of *ADCY3* in each cell split by genotype. N = 186,341 cells

Supplementary Figure 6

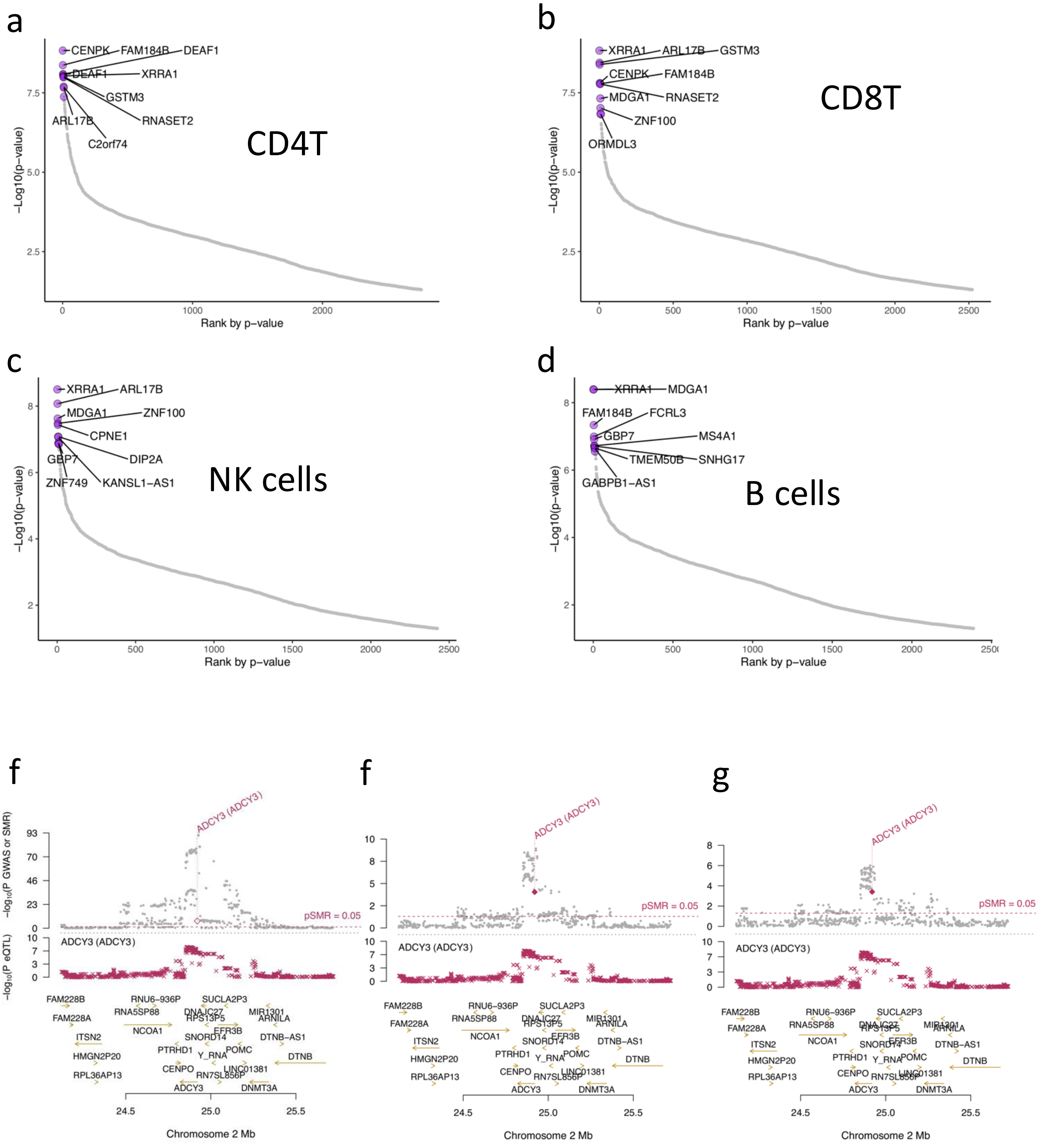

Supplementary Figure 6

(a-d) Top consistent eQTL identified in each cell lineage. (e-g) Locus plot of the SMR analysis for *ADCY3* eQTL vs BMI, *ADCY3* eQTL vs OB1, and *ADCY3* eQTL vs OB2.

Supplementary Figure 7

a

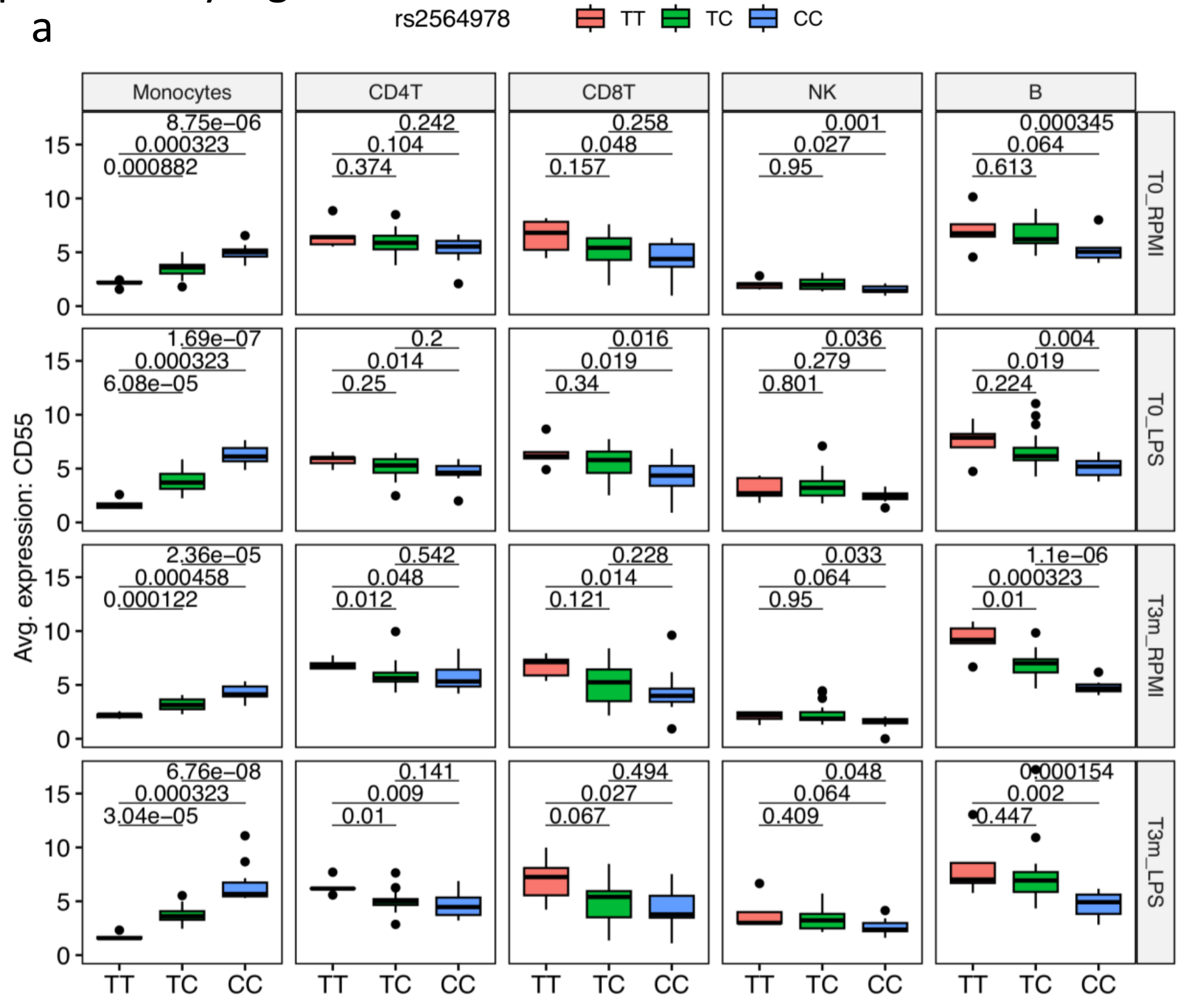

b

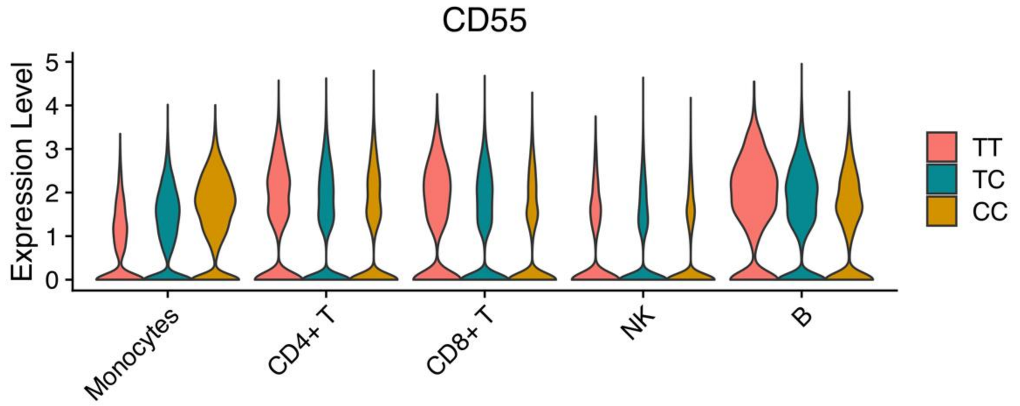

Supplementary Figure 7

(a) eQTL effects of *CD55* locus estimated per condition per cell lineage type (n = 152 samples). In box-pots, whiskers expand to minima and maxima, boxes indicate 25th and 75th percentiles, centers represent medians.

(b) Violin plot displays the eQTL effects of *CD55* locus at single-cell level, n = 186,341 cells.

Supplementary Figure 8

a

	Tissue	Samples	NES	p-value	m-value
	Whole Blood	670	0.256	1.4e-56	1.00
	Thyroid	574	0.328	1.3e-23	1.00
	Adipose - Visceral (Omentum)	469	0.282	1.7e-20	1.00
	Esophagus - Mucosa	497	0.292	1.2e-18	1.00
	Skin - Not Sun Exposed (Suprapubic)	517	0.226	9.8e-14	1.00
	Muscle - Skeletal	706	0.143	4.1e-12	1.00
	Skin - Sun Exposed (Lower leg)	605	0.213	5.2e-10	1.00
	Artery - Aorta	387	-0.228	3.5e-8	0.00
	Spleen	227	0.213	6.7e-8	1.00
	Brain - Cerebellum	209	-0.406	8.7e-8	0.00
	Brain - Cerebellar Hemisphere	175	-0.442	4.5e-7	0.00
	Breast - Mammary Tissue	396	0.195	7.3e-7	1.00
	Esophagus - Muscularis	465	0.167	1.1e-5	1.00
	Nerve - Tibial	532	0.128	1.4e-5	1.00
	Pituitary	237	0.222	2.5e-4	1.00
	Small Intestine - Terminal Ileum	174	0.285	3.7e-4	1.00
	Stomach	324	0.134	1.3e-3	0.941
	Vagina	141	0.250	1.6e-3	0.984
	Prostate	221	0.170	2.8e-3	0.987
	Colon - Transverse	368	0.132	0.006	0.821
	Brain - Cortex	205	-0.145	0.007	0.00
	Testis	322	-0.104	0.007	0.00
	Brain - Nucleus accumbens (basal ganglia)	202	-0.139	0.01	0.00
	Uterus	129	0.226	0.02	0.989
	Cells - Cultured fibroblasts	483	0.0732	0.02	0.00
	Artery - Coronary	213	0.154	0.02	0.810
	Pancreas	305	0.110	0.04	0.481
	Liver	208	0.104	0.04	0.395
	Esophagus - Gastroesophageal Junction	330	0.0778	0.04	0.00500
	Brain - Hypothalamus	170	-0.112	0.05	0.00
	Adrenal Gland	233	-0.143	0.06	0.00
	Adipose - Subcutaneous	581	0.0649	0.06	0.00
	Heart - Atrial Appendage	372	0.0694	0.09	0.00300
	Brain - Spinal cord (cervical c-1)	126	0.145	0.1	0.545
	Brain - Putamen (basal ganglia)	170	-0.0804	0.2	0.00
	Ovary	167	0.0756	0.2	0.101
	Brain - Hippocampus	165	-0.0595	0.3	0.00
	Brain - Anterior cingulate cortex (BA24)	147	0.0684	0.3	0.119
	Brain - Amygdala	129	0.0752	0.4	0.164
	Lung	515	0.0207	0.5	0.00
	Artery - Tibial	584	0.0198	0.5	0.00
	Brain - Caudate (basal ganglia)	194	-0.0272	0.6	0.00
	Cells - EBV-transformed lymphocytes	147	-0.0317	0.6	0.00
	Minor Salivary Gland	144	-0.0483	0.7	0.0290
	Brain - Frontal Cortex (BA9)	175	-0.0141	0.8	0.00300
	Brain - Substantia nigra	114	0.0163	0.8	0.0750
	Kidney - Cortex	73	0.0116	0.9	0.0710
	Heart - Left Ventricle	386	0.00189	1	0.00
	Colon - Sigmoid	318	-0.00224	1	0.00

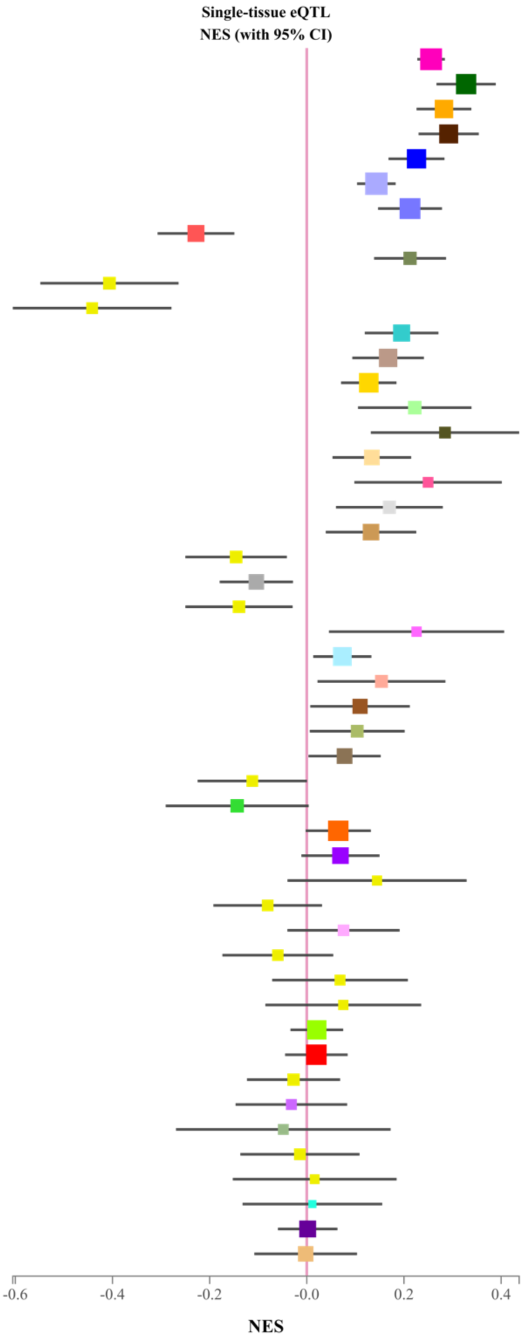

b

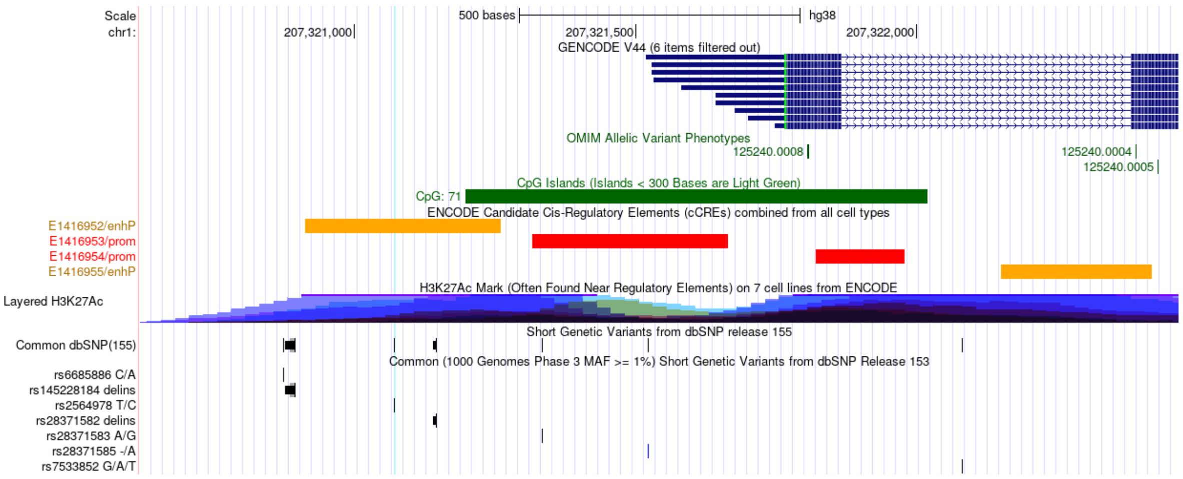

Supplementary Figure 8

(a) *CD55* eQTL effect in GTEx tissue-eQTL results. The data are represented with mean expression +/- SD (95% CI).

(b) *CD55*-associated eVariant is located in a potential regulation element (UCSC genome browser).

Supplementary Figure 9

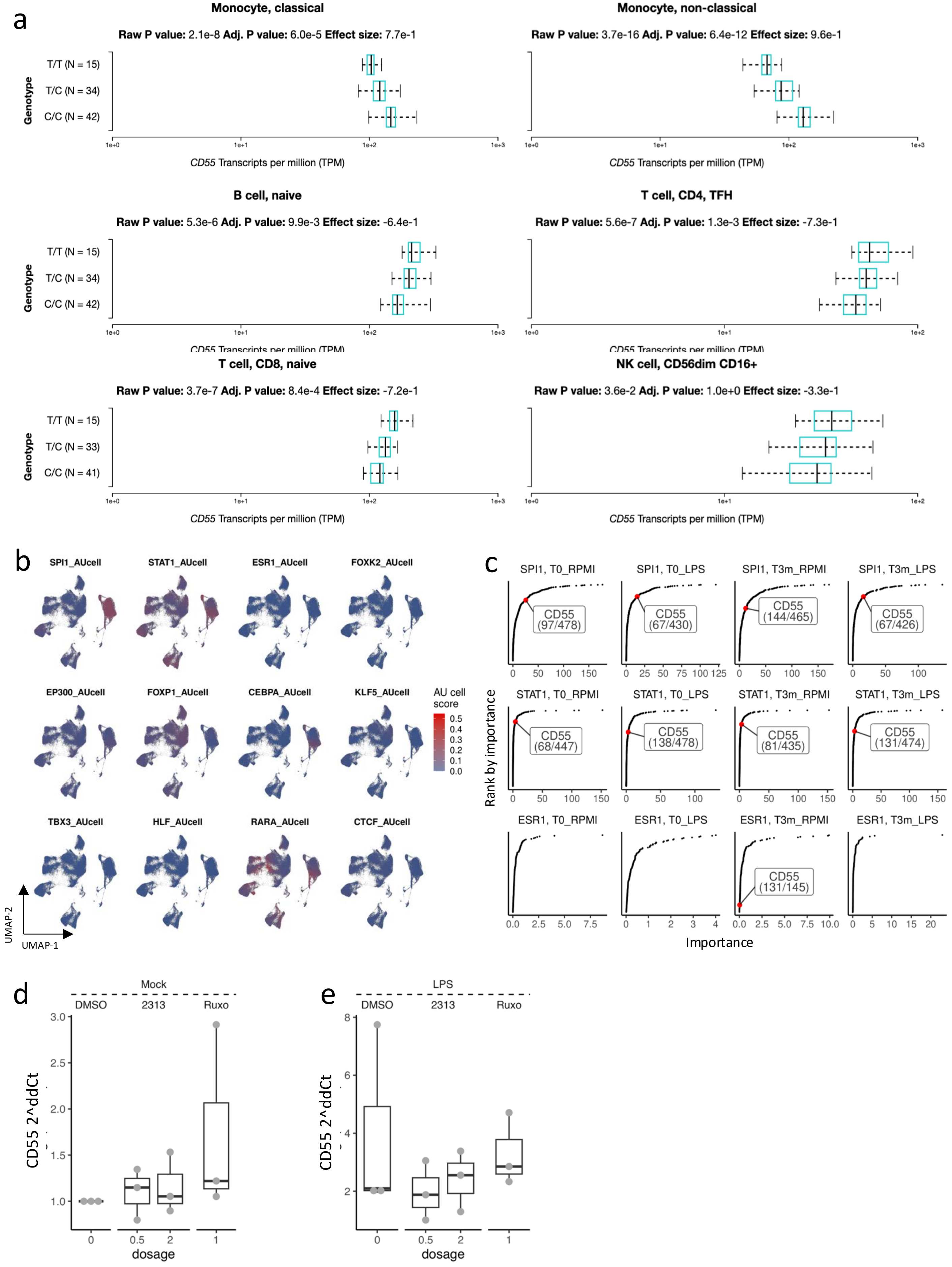

Supplementary Figure 9

(a) eQTL effect of *CD55*-associated eVariant in DICE database which aims to understand the common genetic variations in human disease. In box-pots, whiskers expand to minima and maxima, boxes indicate 25th and 75th percentiles, centers represent medians.

(b) UMAPs shows estimated AU cell score by SCENIC[39] for the prioritized TFs. Each panel represents the AU cell score of a TF and the color indicates AU cell score.

(c) The estimated importance of TF-targets in each TF regulon that is specifically bound to the rs2564978-C allele. Each panel indicates the regulatory capacity of a given TF in one condition. The x-axis indicates the estimated importance score by SCENIC[39], while the y-axis indicates the rank of the regulatory target by the TF. *CD55* was labelled and its rank was included in the labels.

(d) and (e) The inhibition of potential TFs (SPI1 and STAT1) that regulates *CD55* expression in monocytes. 2313 = DB2313. Ruxo = Ruxolitinib. Dosage: numbers indicate micromolar concentrations. In box-pots, whiskers expand to minima and maxima, boxes indicate 25th and 75th percentiles, centers represent medians.

Supplementary Figure 10

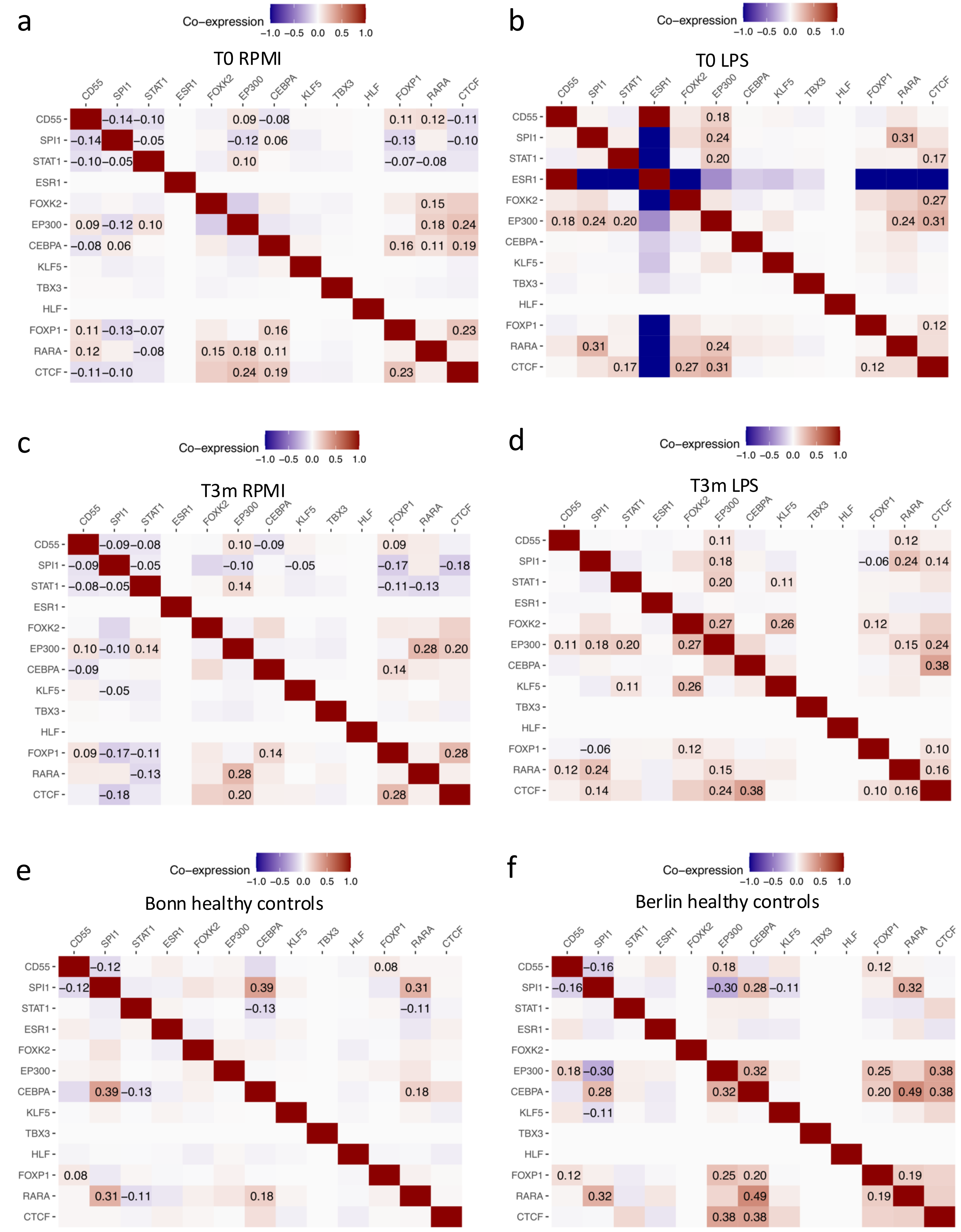

Supplementary Figure 10

Co-expression of *CD55* and prioritized TF genes in the all conditions (a-d) and publicly available datasets (e-f), n = 186,341 cells.

Supplementary Figure 11

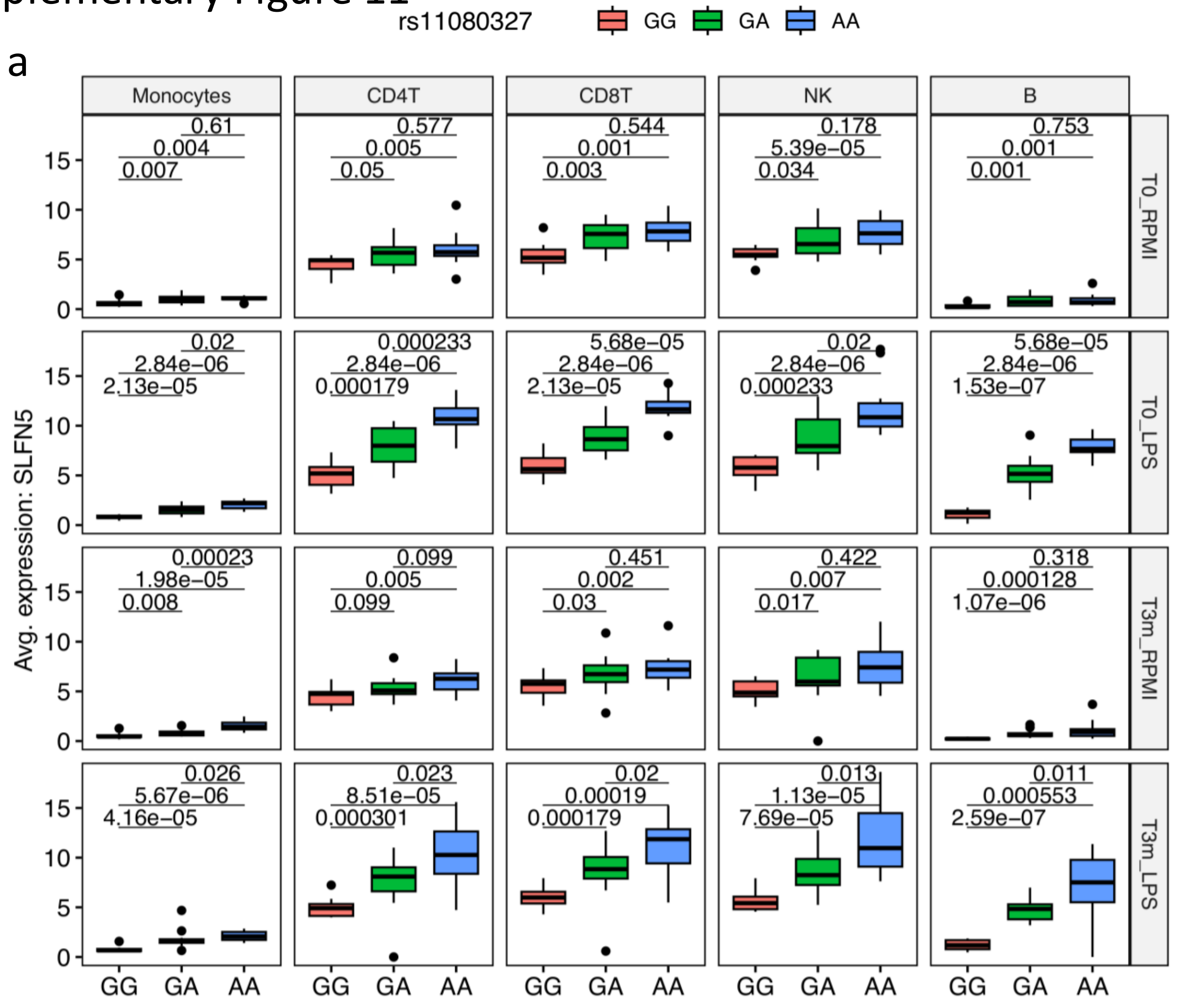

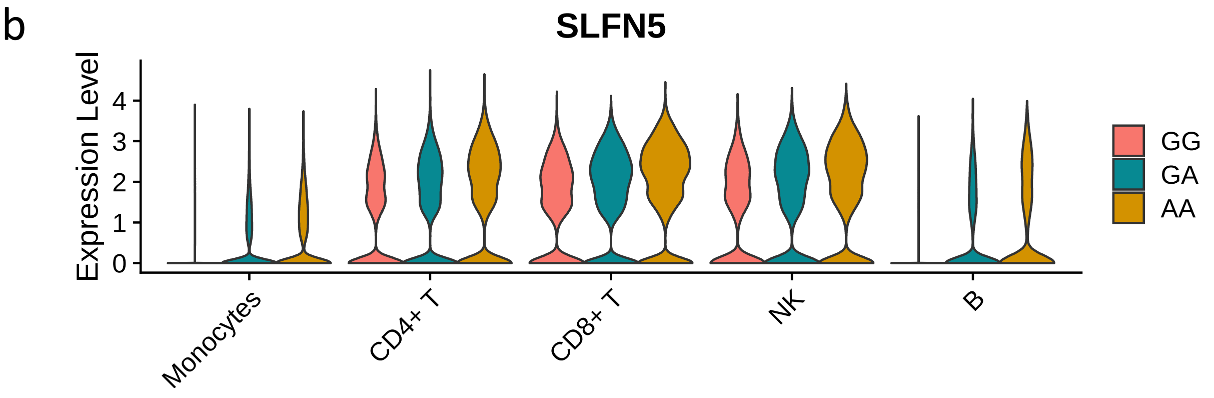

Supplementary Figure 11

(a) eQTL effects of *SLFN5* locus estimated per condition per cell lineage type. The gene expression is compared by two-sided t-test, n = 152 samples. In box-pots, whiskers expand to minima and maxima, boxes indicate 25th and 75th percentiles, centers represent medians.

(b) Violin plot displays the eQTL effects of *SLFN5* locus at single-cell level. N = 186,341 cells.

Supplementary Figure 12

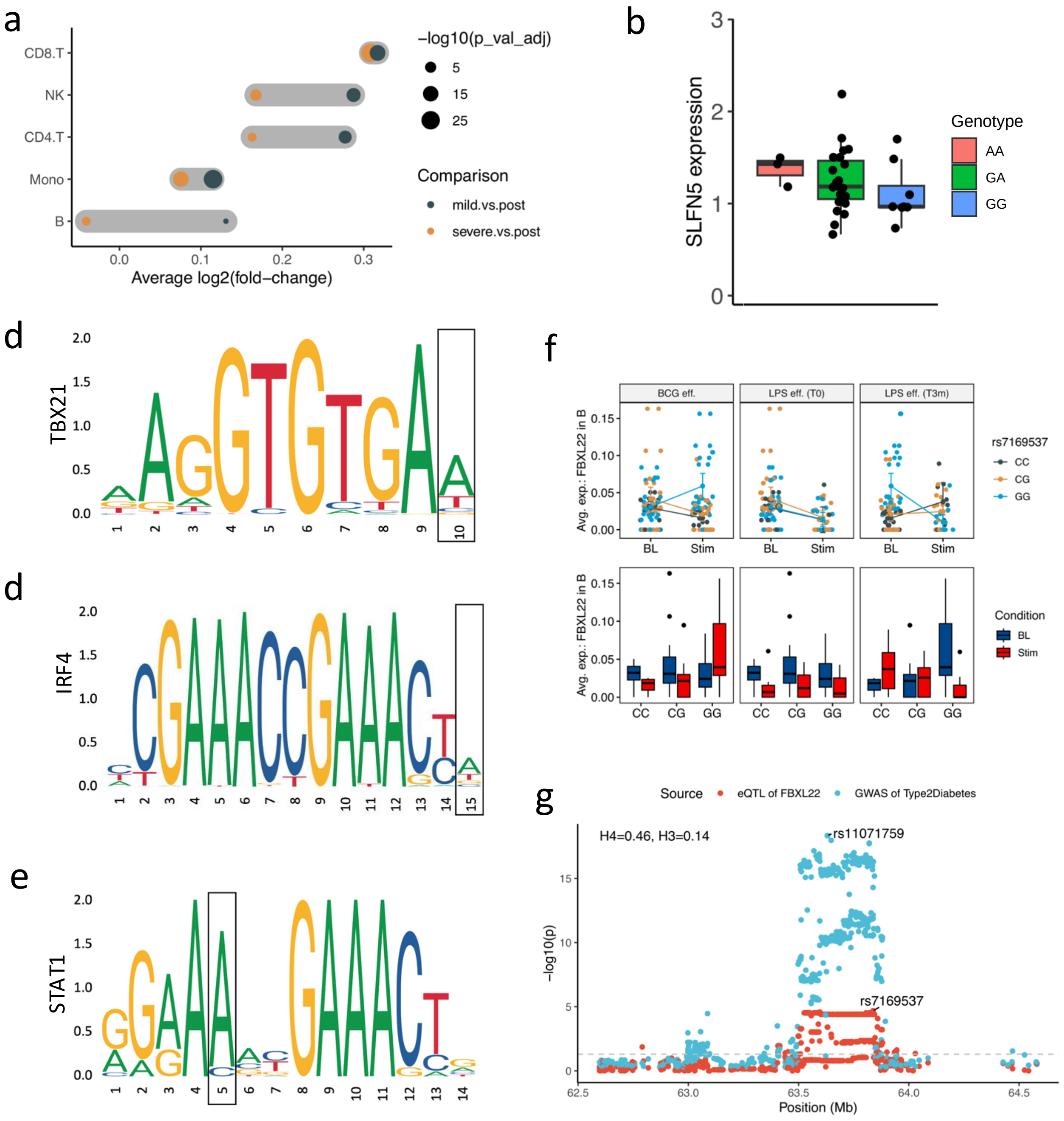

Supplementary Figure 12

(a) Elevated expression of *SLFN5* in hospitalized (mild and severe) individuals compared to convalescent (post) individuals.

(b) Validation of eQTL effect of *SLFN5*-rs11080237 in an independent cohort. In box-pots, whiskers expand to minima and maxima, boxes indicate 25th and 75th percentiles, centers represent medians (two-sided t-test, n = 43 samples).

(c-e) Binding motifs of the prioritized TFs that cover the eVariant associated with *SLFN5* expression upon stimulation. The frame indicates the position of the eVariant.

(f) Response eQTL effects of *FBXL22* gene. There are six panels in three columns. Each columns represents the gene expression alteration upon stimulations from one condition. In the dot plots, the x-axis are treatments, either "BL" or "Stim", and the y-axis is the gene expression level. The dots represent one donor and colors indicates genotypes. Data is presented with mean expression +/- SD (95% CI). The box plots represent the gene expression per genotype with x-axis and y-axis showing genotypes and gene expressions, respectively; the color indicates Conditions which matches the to the top dot plots. Whiskers of box-plots expand to minima and maxima, boxes indicate 25th and 75th percentiles, centers represent medians.

(g) Colocalization of *FBLX22* eQTL and type II diabetes. The x-axis is the genome positions in base-pairs and the y-axis is the p-value transformed by -log10(). The colors of dots indicate eQTL (red) or GWAS (blue). The top SNPs from two summary statistic were labeled and the colocalization H4 and H3 were also labeled (top left corner). The PP4 is estimated by Bayesian colocalization analysis using Bayes Factors.
