# Supplementary material for: Unveiling genetic signatures of immune response in immune-related diseases through single-cell eQTL analysis across diverse conditions
Source: Nat Commun. 2025 Aug 4;16:7134. doi: 10.1038/s41467-025-61192-4 (PMC12322060; doi:10.1038/s41467-025-61192-4)
Supplement: Supplementary file 2 — Description Of Additional Supplementary Files [file 41467_2025_61192_MOESM2_ESM.pdf]

Description of Additional Supplementary Files

File Name: Supplementary Data 1
Description: The TI cQTL effects of identified TI eQTL. The TI eQTLs are estimated by linear mixed model adjusting age and sex and TI cQTLs are estimated by linear model adjusting age and sex.

File Name: Supplementary Data 2
Description: Differentially expression of LCP1 in monocytes from COVID-19 patients. The differentialy expression are estimated using Wilcoxon signed-rank test.

File Name: Supplementary Data 3
Description: Publicly available GWAS summary statistics used in current study.

File Name: Supplementary Data 4
Description: Colocalization results between sc-eQTLs and publicly available GWAS summary statistics.

File Name: Supplementary Data 5
Description: Independent loci estimated from publicly available GWAS summary statistics.
